# Supplementary material for: Prevalence and Haplotypes of Toxoplasma gondii in Native Village Chickens and Pigs in Peninsular Malaysia
Source: Vet Sci. 2023 May 6;10(5):334. doi: 10.3390/vetsci10050334 (PMC10221600; doi:10.3390/vetsci10050334)
Supplement: Supplementary file 1 [file vetsci-10-00334-s001.zip › vetsci-2297408-supplementary.pdf]

Table S1. Descriptions and coding of the variables included in the study as potential risk factors for *Toxoplasma gondii* infection in village chicken and pig farm.

Variables	Description	Coding of the variables
Farming system	What system of farming that was used for raising village chicken and pig?	Village chicken: Free-range/Caged<br>Pig: Open-house/Closed-house
Type of feed	What feed type that was given to the animals?	Commercial (commercially bought feed)/Produced on farm (table scraps, swill, homemade)
Feed storage	Where is the animal feed stored?	Open (open silo/storage)/Close (close silo/storage)
Feeding location	Are there feeders used in the site?	On ground (either all on the floor or some on and some off the floor)/Off ground (individual feeders, bowl, pipeline)
Water source	Where is the source of drinking water for the animals in the farms?	Tap/well
Farm often cleaned	Is it a common practise to clean between batches?	Yes (always, most of the times)/No (rarely, never)
Presence of other livestock	Are there other livestock species in and around (<1km radius) the farm in addition to village chicken and pig?	Yes/No
Presence of cats and/or dogs	Have farmers observed resident and/or stray cats and/or dogs in and around the perimeter of the farm where village chicken and pig are reared?	Yes/No
Presence of rodents	Have farmers observed rodents in and around the perimeter of the farm	Yes/No

	where village chicken and pig are reared?	
Wild animal close contact with studied animals	Is it possible wild animals such as rodents, birds and wild boars come in close proximity, and share feed and water with village chicken and pig in the farm?	Yes/No
Other animals have access to feed and water	Is it possible other wild and stray animals access water reservoirs and feed in the farm?	Yes/No
Rodent control program	Do farmers execute rodent control (rodent proofing of barn and rodent control program) in the farm?	Yes/No
